# The effect of the serum corona on interactions between a single nano-object and a living cell

**DOI:** 10.1038/srep45758

**Published:** 2017-04-06

**Authors:** Yael Dror, Raya Sorkin, Guy Brand, Olga Boubriak, Jill Urban, Jacob Klein

**Affiliations:** 1 Materials and Interfaces Department, Weizmann Institute of Science, Rehovot 76100, Israel; 2 Department of Physical and Theoretical Chemistry, Oxford University, Oxford OX1 3QZ, United Kingdom; 3 University Laboratory of Physiology, Oxford University, Parks Road, Oxford OX1 3PT, United Kingdom

## Abstract

Nanoparticles (NPs) which enter physiological fluids are rapidly coated by proteins, forming a so-called corona which may strongly modify their interaction with tissues and cells relative to the bare NPs. In this work the interactions between a living cell and a nano-object, and in particular the effect on this of the adsorption of serum proteins, are directly examined by measuring the forces arising as an Atomic Force Microscope tip (diameter 20 nm) - simulating a nano-object - approaches and contacts a cell. We find that the presence of a serum protein corona on the tip strongly modifies the interaction as indicated by pronounced increase in the indentation, hysteresis and work of adhesion compared to a bare tip. Classically one expects an AFM tip interacting with a cell surface to be repelled due to cell elastic distortion, offset by tip-cell adhesion, and indeed such a model fits the bare-tip/cell interaction, in agreement with earlier work. However, the force plots obtained with serum-modified tips are very different, indicating that the cell is much more compliant to the approaching tip. The insights obtained in this work may promote better design of NPs for drug delivery and other nano-medical applications.

Rapid progress has been made towards the incorporation of nanoparticles (NPs) in biological systems and nano-medicine, such as drug delivery systems, and diagnostic and therapeutic nano-tools^1^,^2^,^3^,^4^. At the same time, the small size of NPs, which make them attractive in such applications, is also a potential drawback, as they can travel along many pathways in the body and enter almost any tissue, cell or organelle^5^. In consequence, there have been extensive related studies of the cell/nano-material interface, both from applied and basic aspects, alongside the newly emerging field of nanotoxicology^1^,^6^,^7^,^8^. It is well known that as nanoparticles enter the body fluids they are coated by a protein corona^1^,^9^,^10^,^11^ and hence the entity that the cell interacts with is not a bare nanoparticle, but rather a hybrid unit made of the protein corona and the coated particle. The corona’s composition and how it is related to the material and surface of the nanoparticle, the rate of association and dissociation and the lifetime of this coating were comprehensively assessed by Dawson and co-workers^12^,^13^,^14^,^15^,^16^. It is hypothesized that a particle decorated by a corona which contains proteins that are recognized by cell receptors may interact specifically with the cell and trigger an internalization process, while bare particles or particles coated by unrecognized proteins may interact differently^17^. Protein adsorption on a nanoparticle surface has been associated with facilitated uptake by cells for several different materials^18^,^19^, explained by entrance via multiple receptors enabled by the diverse protein composition of the serum^18^. However, due to the different corona compositions on NPs with different properties (such as NP composition, size or shape), some studies demonstrate enhanced nanoparticle uptake for serum incubated NPs^18^,^19^,^20^,^21^, while other studies show the opposite^22^,^23^,^24^,^25^,^26^,^27^.

The prevailing methodology in all the above mentioned studies of nanoparticle-cell interactions is incubation of cells with fluorescently labelled particles in the presence and absence of serum, following by evaluation of the particle uptake by methods of flow cytometry, and/or direct visualization of internalized particles by fluorescence/confocal and electron microscopy. Indeed, the question of cell-nanoparticle interaction has been addressed, so far, through investigation of the uptake process of specific particles^4^,^9^,^28^. However, the direct measurement of the actual forces between a living cell and a nano-object has been much less addressed^29^,^30^,^31^. The molecular force probe (MFP), a device based on an AFM platform but optimized to measure forces, provides the capability to directly examine the interaction between a nano-object and a single cell.

In this study we focus on measuring the interactions between an AFM nano-tip and a living primary cell directly. In particular, we examine the effect on this interaction of exposing the nano-tip to serum, so that serum components may adsorb on it, relative to an identical nano-tip which had only been in contact with a medium containing no serum proteins. To minimize effects arising from the distortion of the cell as a whole by the tip, we used low forces (in the range up to 100 pN) at slow loading rates – far lower than in previous AFM studies of cells^32^,^33^,^34^. With such small forces, and using small tips with radii of about 20 nm, measurements are challenging as the interaction is very local and sensitive to any variation in local composition of the cell membrane or the pericellular matrix. In particular, at such scales local variations in the density of actin filaments and microtubules can affect the local mechanical properties. Fetal bovine serum, which contains more than 3000 different proteins^14^, was used as the environment for the cell and the interacting tip. As far as we know, this study is the first to examine directly the effect of the resulting corona (which we attribute to proteins adsorbed from the serum, as observed earlier for free nanoparticles^13^,^35^,^36^) on the forces between a nano-object (the tip) and a living cell. We probe the cell-nano-object interaction directly, therefore indirect effects of serum, such as its effect on aggregation and adhesion^37^ do not interfere with our measurements.

## Results and Discussion

**Figure 1:**
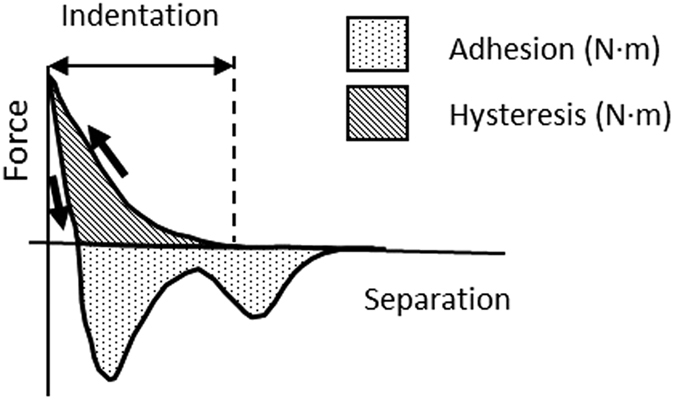
Schematic force profile and the retrieved adhesion, hysteresis and indentation parameters.

Cell imaging and force measurements were carried out using a scanning force-probe device employing very sensitive commercially-available cantilevers. Force-separation cycles were carried out as described in *Materials and Methods.* Each force-separation curve was analysed to evaluate the amount of *indentation*, defined as the vertical distance the tip travelled from the contact point to the trigger point; *hysteresis*, representing the dissipated energy; and *work of adhesion*, as shown schematically in Fig. 1 (and on an actual force curve in the supplementary information (S2)).

**Figure 2:**
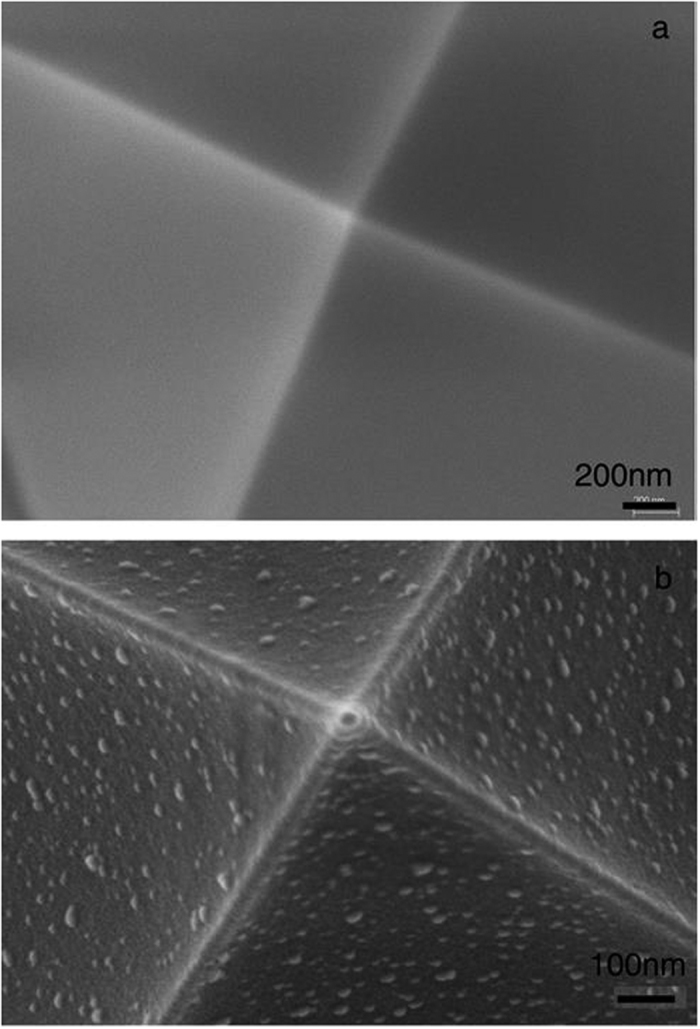
Showing a corona adsorbed on the AFM tip that had been exposed to medium plus serum (ComM, group II), which we attribute to serum protein adsorption (see text). SEM micrographs of (**a**) bare and (**b**) ComM pre-incubated tips. We note that AFM tips that were exposed to medium alone (no serum, as in Group I) showed little difference to bare tips that had not been incubated.

Two groups of experiments were carried out as follows**: Group I**: cells were cultured in complete medium (ComM) containing medium and serum; however one hour before the experiment the ComM was replaced by medium (M) only (containing no serum proteins). **Group II**: cells were cultured in ComM and one hour before the experiment the ComM was replaced by fresh ComM: In order to reveal what might happen to the tip once it is exposed to ComM in Group II, the tip was incubated for one hour in ComM at the same temperature as the measurements (i.e. 37 ^°^C) and then washed, dried and coated for further visualization by SEM. The qualitative difference between a bare and a pre-incubated tip is demonstrated in dry-mode SEM micrographs presented in Fig. 2, showing a corona adsorbed from the serum coating the tip apex together with deposited particles all over the tip surface. We note that while the adsorption takes place from the ComM, the SEM image is taken under dry conditions, implying that the shape of the imaged adsorbed species may differ somewhat from that in solution. The micrograph suggests – for example, the protruding layers on the pyramid edges and vertex - that the corona consists of an adsorbed multilayer rather than a monolayer. The size of the deposited particles – roughly 8–25 nm – is typical of serum proteins^38^ or protein aggregates, and it is thus reasonable to attribute the corona on the tip to proteins adsorbed from the serum, as has been shown for free NPs in several studies^13^,^35^,^36^. It would be of interest to identify these tip-adsorbed proteins explicitly, as may be attempted in future work, though it should be noted that this is far from trivial. Not only is the adsorbance on a single nano-tip minute, but separating tip-adsorbed proteins from those adsorbed on the much larger cantilever to which the tip is attached, which is also immersed in the ComM but which is made from a different material, is especially challenging. Changing the solution one hour before the measurements enabled the cells to stabilize to any changed medium composition; use of fresh medium also ensured that the dish was as free as possible of floating bio-material that might stick to the tip and alter the force measurements.

Prior to force measurements the cells were imaged in order to pick the location at which the force measurements were to be subsequently acquired. The cell imaging also ensured that the chosen cell was indeed well adhered to the petri dish and healthy in appearance. To avoid the possibility that the underlying substrate was affecting the measurement, only high points were chosen for the subsequent force measurements. As we aimed to probe the interaction between the AFM tip and the cell rather than the mechanical properties of the cell, the applied load both during the scanning and the force measurements was kept as low as 100 pN, which is only about 10-fold larger than the amplitude of the noise (ca. ±7–8 pN) in the force-distance profiles. The very soft cantilever chosen for this required application of low-load contact mode (CM), for the cell imaging, as opposed to the frequently-used tapping mode^39^ or regular CM where much higher forces (2–5 nN) are applied^34^,^40^,^41^. However, in contrast to regular CM-imaging that might injure the cells^42^, the low-force CM imaging suppresses cell damage and yet provides high resolution, as was earlier demonstrated by Campbell^43^ and Le Grimellec^44^,^45^. A typical OA (outer annulus) cell imaged by low-force CM is presented in Figure S1 in the supporting information.

**Figure 3:**
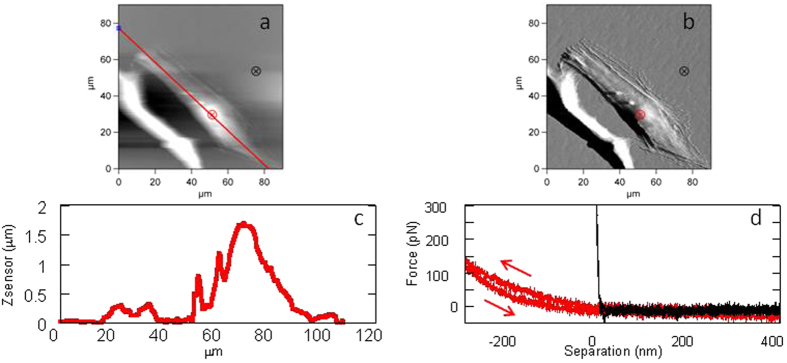
Typical AFM scanning and force profiles of Group I cells i.e. in M only: (**a**) 90 × 90 um scan of an OA cell, height mode; (**b**) scan in error mode; (**c**) cross section of the height along the red line plotted in (**a**); (**d**) Force-approach and force-separation profiles measured above the nucleus (red) and on the petri dish (black), at the points marked by crossed circles in a and b.

A typical force profile measured in medium without proteins (i.e. a bare tip, Group I) is presented in Fig. 3. The force curve taken at a high point as can be seen in the cross section (3c) (taken along the red line in Fig. 3a) is characterized by a relatively large indentation (~300 nm) with no indication of membrane penetration and a small hysteresis between the approach and the retraction curves (red curves in Fig. 3d, taken at the red point in Fig. 3a. This is in contrast with force profiles measured at low points on the cell where the substrate is already felt (not shown) or on the bare substrate (such as petri dish- crossed circle on the surface, Fig. 3a), black curve in Fig. 3d. In this case the indentation is significantly smaller with a linear increase in the force in the contact regime and no hysteresis is observed, as expected from a force profile measured against a hard surface.

**Figure 4:**
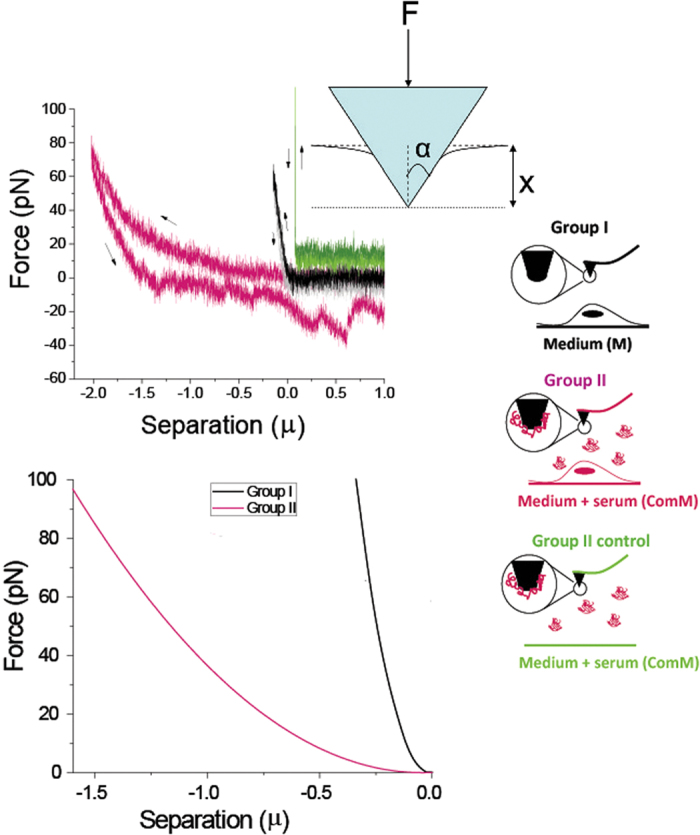
Showing how exposure of the tip to serum (with the associated adsorbed corona attributed to serum proteins, see text) modifies the force profile. Comparison between typical force profiles of the groups I and II schematically described on the right: Group I- no proteins are present, Group II - serum proteins are suspended in the surrounding and coating the AFM tip. (**a**) Shows actual plots for each system, approach and retraction as indicated by the arrows. For group II, approach and retraction on a petrie dish in the presence of serum in the surrounding medium is also presented, (green curve, slightly shifted for clarity), showing that the compressibility of the serum coating on the tip does not account for the large difference between groups I and II. Inset: schematic drawing of a pyramidal tip indenting a half-space made of a stretchable elastic material. The angle of the pyramidal tip is α. The total displacement due to indentation under force F is *x*. (**b**) Shows the plots corresponding to the modes for each group (rather than the means, see text). Each curve was fitted to a parabolic function, the resulted fitted constants of the parabola were extracted and their mode was found (SI).

**Figure 5:**
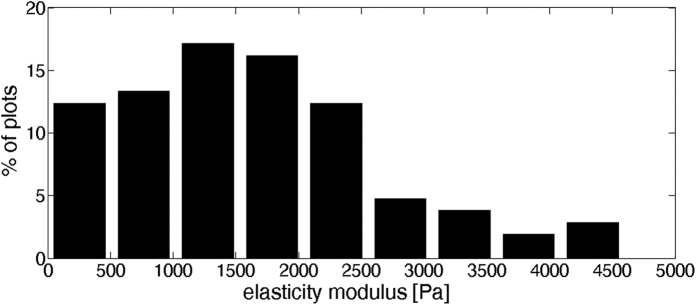
Histogram of Group I elasticity modulus values, showing the skewed (rather than Gaussian) distribution of values.

The clear difference between the two groups is highlighted in Fig. 4. Figure 4(a) shows a representative force profile of each group over a similar range of normal loading (roughly 100 pN). Figure 4(b) shows the most probable force curve plot for each of the groups, as is explained below, based on analysis of 100 plots for group I, and 170 plots for group II (including many different contact points and several different cells for each group). The variation in the obtained force profiles for each of the groups was relatively large. This variation results from imprecise positioning of the tip at the highest point on the cell as a result of thermal drift in the instrument, as well as from natural diversity between the live cells, and cellular movement during the experiment. In particular the cytoskeleton morphology varies over the scale of tens of nm which is the lateral extent of the tip-cell interaction on initial contact^46^. The large variation can be seen in Fig. 5, which shows the probability distribution of the modulus values for group I (bare tip contacting the cell; the values were extracted from the plots as discussed below). A similar variation in AFM-tip/cell interaction was previously reported and attributed to similar factors^30^.

Our analysis focuses on comparing these two cases, group I (bare tip in serum-free medium) and group II (corona-coated tip in complete medium including serum). Figure 4a, where representative profiles of the two groups are shown, clearly demonstrates that all three measurable parameters, namely, range of indentation, extent of hysteresis and work of adhesion are significantly smaller in Group I experiments (absence of serum proteins, black profile and associated cartoon) relative to Group II measurements (serum proteins present, see cartoon). In Fig. 4b are shown the approach curves based on fitting (as described below) and averaging all profiles within each group (taking the mode rather than the mean, due to the skewness of the modulus distribution, see SI). These prominent differences between the two cases, with and without serum addition, are very clear above any variation in the data, as further considered below. We emphasize that the presence of serum coating on the tip in itself does not account for the large differences between the two groups, as can be clearly seen in the control plot obtained for a serum-corona coated tip pressing on a petri-dish bare surface, shown in green. In this control plot no hysteresis is observed. As a typical reference petri-dish curve for Group I is shown in Fig. 3d, we do not include it again in Fig. 4a for clarity.

How can we interpret the large differences in these force data plots? The mechanical behaviour of the cell upon deformation by the AFM tip can be understood as follows: indentation of a live cell induces deformations in the cell membrane and cytoskeleton. During indentation, the membrane suffers stretching and bending deformations. As the membrane itself is not smooth and tense, it may stretch under the AFM tip without exhibiting a noticeable elastic force^30^. The force required to bend the membrane may also be neglected due to its low bending rigidity^30^,^47^. We are then left with the elastic deformation of the cytoskeleton, and any adhesion effects (arising from chemical affinity between the tip and the membrane). It is well established that the elastic response of cells to indentation is mostly due to the cytoskeletal actin network^48^. The membrane may, however, experience an adhesive attraction to the AFM tip indenting it. If we assume that the indenter-cell contact area and the associated adhesion energy increase progressively during the indentation (as the indenter has a pyramidal shape, see Fig. 2 and schematic inset to Fig. 4A), then the externally applied force *F(x)* that is required to indent a cell (in the x direction) is generally determined by a combination of an elastic force exerted by the cell cytoskeleton and an adhesive force due to cell-membrane/AFM-tip adhesion. A detailed model has been derived by Sirghi *et al*.^30^ for the indentation of a soft, adhesive material with a pyramidal indenter:


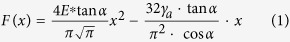


Here E* is the indenter/sample reduced Young’s modulus (for indenters much stiffer then the sample, E* = E/(1−*v*^2^) where E is the Young modulus of elasticity and the Poisson ratio, *v*, is considered to be 0.5 for cells), α = 35^°^ is the pyramid angle of the AFM tip and *γ*_*a*_ is the adhesion energy^30^. The model described above can readily be used to fit our data. Fitting to the Group I data, bare AFM tip in serum-free medium, is done automatically with a custom written Matlab code for all 100 separate Group I profiles (SI), and from the best fit we extract the values of the modulus and adhesion energy. The probability distribution of the modulus values for group I is shown in Fig. 5 (and is seen to be skewed, as noted earlier). We find maximum probability values of E* = 1.3 kPa, and *γ*_*a*_=5.6·10^*−5*^*J*·*m*^*−2*^, very comparable to previously reported values^30^ (that were obtained for a different cell type).

**Figure 6:**
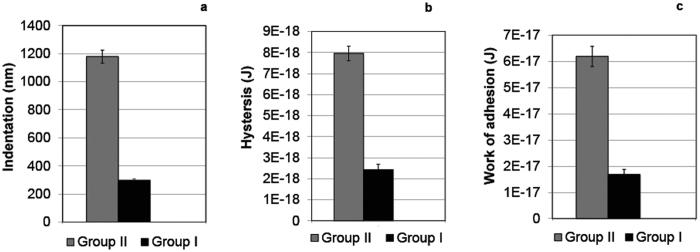
Statistical results of (**a**) Amount of indentation (nm), (**b**) hysteresis (J) and (**c**) work of adhesion (J) for Group I measured in M (grey) and Group II measured in ComM (black). The data are presented as mean ± standard error. The results presented for each groups are based on a 100 or more independent force profiles per group (*Materials and Methods*).

The average modulus of a cell depends on the particular cell type. While the modulus value can vary locally when the cell is probed with a sharp tip, the average value taken over many cells and many contact points should be similar and constant, within the scatter, for that given cell type. As the modulus is an internal cellular property that depends largely on the cytoskeleton structure, it should not be affected by tip coating such as the serum protein corona. As all cells were cultured under identical conditions, those incubated in serum and those without serum during the measurements should have the same overall modulus; indeed, it has been shown^49^ that the presence or absence or serum proteins does not affect the overall cell modulus over periods shorter than 3 days. We thus extracted the effective modulus E* = 1.3 kPa of our cells from the Group I results as noted above, and attempted to use this value for fitting the data of group II, as it appears in Fig. 3(B), to Eq. 1 with a single fitting parameter *γ*_*a*_ (we substitute a value E* = 1.3 kPa into Equation (1).) However, there is no value of the adhesion energy which, taken together with this cell modulus, can fit the observed force profiles. This implies that the picture suggested in the inset to Fig. 3(A) and the corresponding Eq. (1), cannot represent what is happening when proteins are present both on the tip as well as in the surrounding medium. Moreover, we have compared values of: 1. average indentation 2. amount of hysteresis and 3. work of adhesion. These average results (based on over a hundred profiles, *Materials and Methods*) are presented as histograms in Fig. 6, and in Table S1 in the SI. Comparing groups I and II, the average values of the three parameters are clearly very different, differences which remain highly significant even when the scatter is accounted for (p < 0.001 in the Kruskal-Wallis test, see also *Materials and Methods*). While the average indentation, amount of hysteresis and the work of adhesion measured in group II are 1179 nm and 7.96 × 10^−18^ J and 6.18 × 10^−17^ J respectively, the values for Group I are about 3–4 times smaller. The variance of the results is considered in more detail in *SI*. The results of all three parameters thus indicate consistently that the presence of serum proteins completely alters the tip-cell interaction.

**Figure 7:**
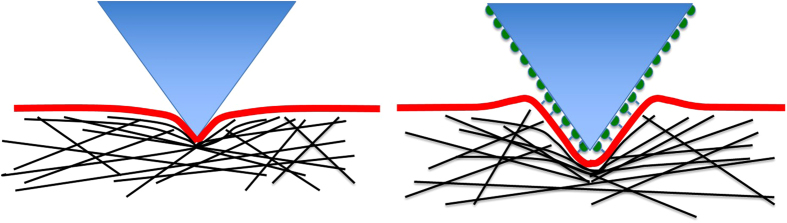
A schematic representation of the engulfment mechanism which results in the differences between indentation by a bare tip, left, and a corona–coated tip, right. For the bare tip the elastic forces (due to deformation of the cytoskeleton elements, shown as black lines) act together with weak forces arising from the membrane adhering to the tip, left. For the corona coated tip, right, its engulfment by the cell membrane results in a substantial attractive force arising from corona-membrane recognition – specific interactions are schematically indicated - which largely offsets the elastic repulsion due to deformation of the cytoskeleton.

How can all these observations on Group II (where the tip is exposed to serum and its corona is attributed to proteins adsorbed from the serum^13^,^35^,^36^, as discussed earlier), namely the incompatibility with the model of Eq. (1), and the very large indentation, hysteresis and adhesion (relative to Group I where the tip is bare), be explained? As the protein-decorated tip contacts and indents the cell, the cell undergoes a strikingly-large deformation with little resistance. That is, the tip continuously presses on the cell without reaching the trigger force, which is only reached after a deep indentation. We stress the difference in the length-scales of the indentations involved - of order of a micron for group II rather than of order of 200 nm for group I. We attribute this as follows: Since, as earlier noted, the effective cell elasticity as a whole (essentially its modulus E*) is due to the cytoskeleton and is unaffected by the corona, one would expect a large elastic resistance F_elastic_ to such a deformation (to depth *x*) of the cell. If we assume that the modulus E* is unchanged with extent of indentation^50^ we may approximate this by taking just the first term in Eq. (1) which dominates at such large indentations since it varies as *x*^2^ while the offsetting adhesion term varies as *x*. For a deformation *x* = 1 μm, this yields an expected resistance force F_elastic_ = 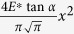 ≈ 6.5 × 10^−10^ N. From Fig. 3(B) however, the actual force F required for a penetration of 1 μm is only ca. 0.4 × 10^−10^ N. The difference between the force F_elastic_ required for a 1 μm deformation of the cell and the actual force needed for such a penetration by the corona-coated tip is thus ca. 6 × 10^−10^ N. In other words, an *attraction* between the tip and the cell of that magnitude (ca. 6 × 10^−10^ N) must have occurred to offset the repulsion that would have been expected from the elastic deformation for such an indentation. We attribute this attraction to the engulfment by the cell membrane of the (corona-coated) AFM tip, and/or the beginning of formation of a pit which is associated with a large local change and rearrangement of the cytoskeleton as happens in several endocytosis mechanisms. e.g. Caveole or Clatherin mediated endocytosis^51^,^52^. This is illustrated schematically in Fig. 7. Clearly complete endocytosis (as would be the case for a small NP^31^) cannot occur, since the AFM tip – whose footprint on the cell surface increases progressively as it indents the cell – cannot be ‘swallowed up’ by the cell.

**Figure 8:**
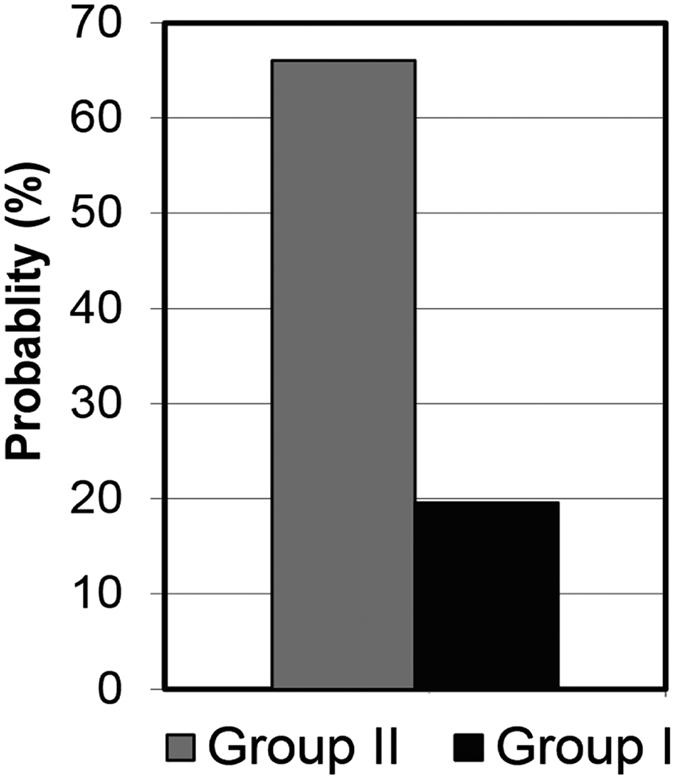
The probability of the formation of specific bonds increases in the presence of free proteins in the medium. This is shown by the frequency of the appearance of multi jump-out peaks pattern describing specific rupture and unfolding events during the retraction curves in Group I measured in M (grey) and Group II measured in ComM (black).

The above observations obtained from the load curves that show a striking difference in the tip-cell interactions between the two groups are in line with the results of the work of adhesion derived from the unloading curves, as summarized in Fig. 6c. Long-ranged pull-off forces resulting in a large work of adhesion as demonstrated in Fig. 4 (pink curve) are related to bonding events such as tethering of receptors to ligands, and clamping of a trans-membranal protein or any other biomacromolecule by the tip^53^,^54^. In addition, simple nonspecific adhesion arising from van der Waals interaction may be playing a minor role. Multi jump-out peaks in a “sawtooth” pattern observed in the unload curves indicate serial rupture of specific bonds or unfolding of macromolecules before full separation between the tip and the cell is retrieved. The rupture force, as observed in the unloading curves, is several tens of pN which is in agreement with specific ligand-receptor data given in the literature^55^,^56^. The probability of these processes increases dramatically in a ComM environment (Group II) due to the presence of many proteins which may bridge the tip to the cell surface. Based on the above, the frequency of the appearance of multi-peaks pattern during the retraction of the tip is expected to be much higher in Group II. This is indeed the case as explicitly shown in Fig. 8. Additionally, the large adhesion shown in Group II is consistent with the large indentation since, on geometrical grounds, the contact area of the tip with the cell surface varies as the square of the indentation enabling the formation of additional specific bonds. Moreover, due to the large indentation the tip spends a longer time in contact with the cell, which allows the formation of these specific bonds contributing to the increase in work of adhesion. We note that the only other AFM study we know of that compared surface-cell interactions in serum containing and serum free media utilized much larger, colloidal probes, of 8–10 μm diameter and demonstrated a twofold increase in the adhesion force between microspheres and cells in the presence of serum^57^.

The interactions measured in this study, particularly as the corona-coated nano-tip makes initial contact with the cell, deforms the cell membrane and indents the cell, are attributed to offset of the elastic deformation force by attraction arising from cell-membrane engulfment of the indenting tip, and are thus related to nanoparticle endocytosis. We may then ask: why do we not observe any net attractive forces on *approach*, but only upon separation? A clue to this is provided by a recent study of endocytic forces on gold NPs^31^ Here the attractive endocytic forces on a 20 nm-diameter NP do not exceed ca.10 pN until the NP has been invaginated into the cell by about 10–15 nm. In the case of our 20 nm-diameter AFM tip, this would suggest that on initial contact and tip indentation of the cell surface by a comparable amount, the endocytic attraction on it would also be of that magnitude, ca. 10 pN (precise comparisons may not be warranted because of differences in NP/tip material and cell types in the two studies). This is within the noise level of our indentation vs. force traces (ca. 10 pN, see force profiles in Figs 3 and 4A). At higher indentation of the cell surface, we believe that the elastic repulsion arising from further cell distortion swamps the initial endocytic attraction, and increasing subsequent indentation proceeds as discussed earlier, with attraction due to engulfment of the corona-coated tip largely offsetting the repulsion due to cell distortion.

A final point concerns the possible variation of composition of the corona on the AFM tip (attributed to adsorbed serum proteins^13^,^35^,^36^) with time of exposure to the serum. As considered in earlier studies of serum protein adsorption on NPs^35^,^58^, this composition may change with time due to the competition between protein concentration and the on/off rates for adsorption of different serum proteins. In our study the force-distance profiles were taken after the AFM tips were exposed to serum for different times, as a single run in which several cells and contact points were measured could take up to three hours. If indeed the corona composition was changing in this time and so resulting in somewhat different cell-tip forces, this may account for some of the scatter in our profiles.

In summary, we have examined directly the force between a nano-object – an AFM tip with a 20 nm extremity radius – and a living cell, both between a bare tip in serum-free medium, and also in the presence of a protein corona adsorbed onto the tip from serum in the surrounding medium (and attributed to adsorbed serum proteins^13^,^35^,^36^). This emulates the corona that rapidly coats all NPs entering the body before they enter into cells by endocytosis. Our results show that as the bare tip progressively indents the surface, its interactions are dominated by a repulsion arising from the distortion of the cell, with an effective modulus (and membrane adhesive energy) similar to earlier studies. In striking contrast, when the corona-coated tip indents the cell surface it experiences a strong attraction which offsets most of the repulsion due to the cell distortion, resulting in a much more compliant behaviour, where a given force on the tip leads to a much larger cell indentation, adhesion and hysteresis relative to the bare tip. We attribute this attraction of the corona-coated tip to partial engulfment of the tip by the cell membrane, a process driven by the recognition of the membrane by the serum-protein corona, and akin to the initial stages of endocytosis. This study, despite utilizing an AFM tip and not a nano-particle, advances the understanding of the effect of corona-driven forces on NP uptake. It demonstrates unambiguously that examining cells in the absence of serum or interstitial fluids, does not provide physiologically relevant information on membrane-nanoparticle interactions. Understanding how nanoparticles interact with cells is important for the design of nanoparticles for biological applications, such as nano medicine. Thus, modifying nano-tip surfaces so as to reduce or increase the ‘softness’ of their interactions with living cell surfaces, as in the present study, may reflect directly on the cellular uptake of similarly surface-modified nanoparticles in living organisms.

## Materials & Methods

### Materials

Dulbecco’s modified Eagle medium (DMEM, Gibco, No. 22320), antibiotic-antimycotic solution (penicillin 10,000 U/ml; streptomycin 10 mg/ml; amphotericin 250 μg/ml, Gibco, No. 15240–062) and foetal bovine serum (FBS)(Gibco, No. 10106–185) were obtained from Ivitrogen (Paisley, UK). Collagenase type I, EC 3.4.24.3 (No. C0130), protease type XIV (No. P5147), DMEM-low glucose without sodium bicarbonate (No. D5523), 4-(2-hydroxyethyl)−1-piperazineethanesulfonic acid (HEPES) and glycerol were purchased from Sigma-Aldrich (Pool, UK).

### Cell culturing (see *SI*)

Primary fibroblast-like cells from the outer annulus (OA) of bovine caudal intervertebral discs from 18–24 month steers were obtained from a local abattoir within 2 hours of slaughter^59^ and were isolated by standard enzyme digestion. The isolated cells were frozen and thawed upon request. Each thawed sample was seeded and cultured in complete medium (ComM) based on, DMEM-low glucose without sodium bicarbonate and with 10% FBS in an incubator without CO_2_ supply at 37 °C.

### Atomic Force Microscopy (AFM)

#### Instrument and Measurements

Cell imaging and force measurements were carried out with a MFP-3D SA (AFM) instrument (Asylum Research, Santa Barbara, CA). Both scanning in contact mode (CM) and force measurements were done using a silicon nitride V-shaped 200 μm long cantilever having a nominal spring constant of 0.02 N/m with a pyramidal silicon nitride tip and a nominal tip diameter 20 nm (TR400, Olympus). Prior to each experiment the cantilever spring constant was determined accurately. This was done by immersing the tip into a petri dish with the appropriate medium at 37 °C, recording force plots for calibration of the optical lever sensitivity and then the spring constant was obtained by applying the thermal method^60^ (see *SI*). Throughout all the experiments the temperature was controlled and measured by a commercially available accessory, Petri Dish Holder and Heater from Asylum Research with 0.05 °C accuracy as stated by the manufacturer. The chip and the chip holder were irradiated in a UV-Ozone device prior to use. A full force profile/cycle consisted of a compression curve, collecting the change in the cantilever deflection as the tip moved towards the cell, until it reached a predetermined load (refers as trigger-mode force measurement) and a decompression curve monitoring the deflection change as the tip moved away from the cell until it was fully retrieved. Several points on each cell were measured, with several repeated measurements at each spot. The approach velocity was 750 nm/s and the pre-determined loading force (trigger point) was about 100 pN. As a control prior to each cell indentation, a force curve on a bare petri-dish surface was obtained for reference, for each of the measurements performed.

#### AFM sample preparation

The cells were seeded and cultured in an uncoated 60 mm petri dish for 5–10 days and used before reaching confluence. The dish was directly transferred from the incubator to a 37 °C preheated petri dish holder mounted on the AFM base/stage (see *SI*).

#### Data analysis

The raw data of force measurements collecting the deflection of the cantilever versus the z-piezo displacement were converted to force versus separation according to well-known procedures^61^,^62^ (see *SI*). Each force-separation curve was analyzed to evaluate the amount of indentation, hysteresis and work of adhesion as shown schematically in Fig. 1. An actual plot that was automatically analysed by our custom written software is shown in supplementary figure S2.

#### Statistical analysis

Due to the intra-cell variation the trigger-mode force profiles were repeated 15–20 times for each cell. In each group 5–9 cells were examined; however we tested no more than 2–3 cells per dish in order to limit the time the cells remained on the AFM stage.

A special algorithm written in Pyton was used to calculate all the measurable variables for each of the curves i.e. indentation, hysteresis and work of adhesion. Any abnormal curve or value was discarded. The averages, standard deviation and standard error were calculated for each group and each measurable variable.

The data were tested for statistical significance difference in order to evaluate if the presence of serum proteins have an effect on the measurable parameters. First, the data were tested for homogeneity of variances between the groups using Bartlett’s test. The results showed that variance within all three parameters varied significantly across the different groups (p < 0.0001, i.e. the null hypothesis of equal variances was rejected). Second, based on the Bartlett’s test results, the Kruskal-Wallis test was applied to determine if there is a statistically significant difference between the three groups. In all tests a significance level of α = 0.05 was chosen. In addition the co-variance (COV) for each variable of each group and within one cell (intra-cell COV) was calculated.

### Scanning Electron Microscopy (SEM)

AFM tips images were obtained using a field emission Ultra 55 high resolution scanning electrons microscope at acceleration voltage of 3 kV and a sample- to-detector distance of 4 mm. The serum-proteins incubated tips were immersed for one hour in serum containing medium, washed and dried by nitrogen flow and then coated with two thin Chromium layers of 2 and 3 nm at two directions. The bare tip remained uncoated. As a separate control to ensure the Chromium-coating was not introducing artefactual structures on the tip, SEM micrographs of Chromium-coated bare tips were obtained and showed them to be identical to the non-Chromium-coated bare tips.

## Additional Information

**How to cite this article:** Dror, Y. *et al*. The effect of the serum corona on interactions between a single nano-object and a living cell. *Sci. Rep.* **7**, 45758; doi: 10.1038/srep45758 (2017).

**Publisher's note:** Springer Nature remains neutral with regard to jurisdictional claims in published maps and institutional affiliations.

## Supplementary Material

Supplementary Information
