# Supplementary material for: The effect of the serum corona on interactions between a single nano-object and a living cell
Source: Sci Rep. 2017 Apr 6;7:45758. doi: 10.1038/srep45758 (PMC5382918; doi:10.1038/srep45758)
Supplement: Supplementary Information [file srep45758-s1.doc]

**Supporting information for:**

**The effect of the serum corona on interactions between a single nano-object and a living cell**

Yael Dror, ^1, 2^ Raya Sorkin,^1^ Guy Brand^1^, Olga Boubriak,^3^ Jill Urban,^3^ Jacob Klein.^1,2*^

^1^ Materials and Interfaces Department, Weizmann Institute of Science, Rehovot 76100, Israel; ^2^ Department of Physical and Theoretical Chemistry, Oxford University, Oxford OX1 3QZ, United Kingdom; ^3^ University Laboratory of Physiology, Oxford University, Parks Road, Oxford OX1 3PT, United Kingdom.

^*^Corresponding author: Jacob Klein, Materials and Interfaces Department, Weizmann Institute of Science, Rehovot 76100, Israel; tel: +972-8-9343823; fax: +972-8-9344137 [Jacob.klein@weizmann.ac.il](mailto:Jacob.klein@weizmann.ac.il)

**Materials & methods**

**Cell culturing**

Primary outer annulus (OA) cells were isolated by standard enzyme digestion from bovine caudal intervertebral discs obtained from 18-24 month steers within 2 hours of slaughter (1). Briefly, the outer third of the annulus was dissected from the upper 5 caudal discs, sliced and digested in DMEM, supplemented with antibiotic-antimycotic solution (1% v/v). Osmolarity of the DMEM was increased by adding a solution containing 5M NaCl and 0.2M KCl to the in-vivo osmolarity of 380 mOsm. The tissue was digested at 37^o^C in presence of 0.1% protease type XIV (w/v) for 4 hrs followed by collagenase type I digestion (0.1% collagenase type I, w/v) for 18 hrs. The digest was filtered through a cell strainer (100 µm pore size) to remove non-digested tissue. The filtrate was centrifuged at ~750 g for 5 min, the supernatant removed and the cell pellet resuspended in DMEM and recentrifuged to remove the excess collagenease. The cells were again resuspended in DMEM and an aliquot taken for measurement of cell number and of viability using trypan blue (1). Finally the cells were centrifuged, resuspended in a freezing medium containing 90 % foetal bovine serum (FBS), and 10% glycerol, frozen frozen according to the Sigma cryopreservation protocol (<http://www.sigmaaldrich.com/life-science/cell-culture/learning-center/ecacc> handbook/cell-culture-techniques-12.html#Protocol%207) in cryo-vials at a concentration of 2million cells/ml) and stored at -80^o^C until used.

For each experiment the cells were thawed within 2-3 min in a water bath set to 37^o^C and cultured in a complete medium (ComM) containing DMEM-low glucose without sodium bicarbonate supplemented with 10%FBS and 1% antibiotic-antimycotic; pH was adjusted to pH7.4 using NaOH and pH maintained with 25mM HEPES. The cells were seeded in 60mm petri dishes at 0.025 million cells·cm^-2^ and cultured in an incubator without added CO_2_ supply at 37^o^C.

**AFM**

*Instrument and Measurements*

The spring constant of each cantilever was determined by using the thermal method (2). This method is based on acquiring a force profile against a hard surface (freshly cleaved mica) to determine the inverse optical lever sensitivity (InVOLS) and measuring the resonance frequency of the cantilever. By applying the equi-partition theorem the spring constant can be determined.

The temperature of the petri dish holder mounted on the AFM stage was controlled by an environmental controller; the holder was pre-heated to 37^0^C before each experiment. Using this setup, perturbations in temperature were minimized during the entire experiment. Moreover, since the cells were cultured in a CO_2_ free incubator and in a medium containing HEPES and without bicarbonate as described above, there was no requirement to alter medium composition for measurements on the AFM stage as often done and the cells remained in their original culture medium and pH for the duration of the AFM manipulations.

In all cases, after the dish was brought to the AFM stage the tip was introduced into the petri dish for 30-60 minutes to allow the system to stabilize before starting the experiment.

*Data analysis*

Raw data of a force cycle were given by the deflection of the cantilever versus the Z-piezo displacement. The conversion of the raw data to force versus separation (between the tip and the sample) was done as follows: (1) the force was calculated using the Hook’s law (*F=Kx*), where *K* is the spring constant and *x* is the cantilever deflection; (2) The zero force was determined by the baseline measured at large tip-sample separation where no interacting forces are experienced and hence the deflection is constant. The data were offset accordingly; (3) The separation was calculated by adding the piezo displacement, Δz, to the cantilever deflection, *x*, and defining the contact point as the zero separation. The contact point was determined visually as the point where the deflection starts to increase monotonically. This rough estimation is justified by the fact that any non-contact repulsion (both electrostatic and steric) is likely to be negligible due to the high ionic strength of the medium and the short range of the steric interaction.

**High resolution imaging of cell in low-force contact mode (CM)**

A typical OA cell imaged by low-force CM is presented in Figure S1 showing the height (a) and error (b) signals. The high pseudonucleous and the well-spread lamelapodium are clearly observed together with the well defined inner structure of the cytoskeleton showing alignment of the fibres along the long axis of the cell.

**
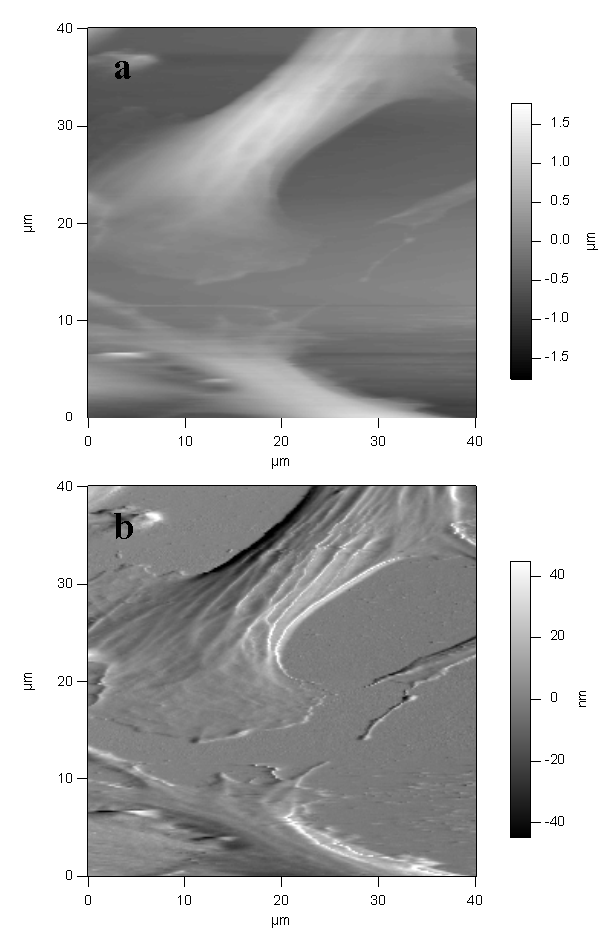
**

**Figure S1**: OA cell imaged in contact mode at 0.5 Hz scan rate and load of 52.64 pN (set point) (a) height, (b) error mode.

**The variances of the statistical results**

It is appropriate to consider the origins of the variation in the values of the indentation and hysteresis measured, as indicated by the values of the co-variance (COV) for these parameters (Table S1). The COV values in all 3 groups are around 30-40%, a wide distribution typical of living cells, (3) arising from the variability of different cells in the same dish, which is expected to be even more pronounced in the case of primary cells. The COV of the results within one cell (intra-cell COV) is also relatively high (between 20-30%), in contrast to typical values of around 6% reported in other works that had used bead-terminated tips (3). This emphasizes the sensitivity of our measurements due to the low trigger point and the nanometric size of the tip: Any small change in the tip position resulting from thermal drift, breathing or movement of the cell (or of the proteins near the contact region) can modify the forces. This complexity is usually averaged out when using a bead-terminated tip. For example, a 10μm diameter bead indenting 0.5μm interacts with a cell-surface area of about 16 μm^2^ whereas with the same indentation a 35^0^ conical tip (see fig. 1) would sample an area of at most ca. 0.7 μm^2^. Nevertheless, the overall COV and the statistical test indicate that the differences between the 3 groups are clearly significant, substantially exceeding the intra-cell variance. The COV of the work of adhesion is higher but the origin of this distribution arises from arbitrary events that may or may not take place as discussed below.

**Table S1**: Values of the measurable variables in the different groups

|  | **Group I** | **Group II** | **Group III** |
| --- | --- | --- | --- |
| Indentation (nm) | 297 | 1279 | 676 |
| STDV | 114 | 554 | 236 |
| COV | 38 % | 43.3 % | 35 % |
| Standard error | 12 | 39 | 15 |
| Hysteresis (J) | 2.44E-18 | 9.22E-18 | 3.65E-18 |
| STDV | 2.42E-18 | 4.76E-18 | 1.34E-18 |
| COV | 99 % | 52 % | 37% |
| Standard error | 2.56E-19 | 3.37E-19 | 8.37E-20 |
| Work of adhesion (J) | 1.52E-17 | 6.79E-17 | 2.22E-17 |
| STDV | 1.68E-17 | 5.50E-17 | 2.65E-17 |
| COV | 110% | 89% | 119% |
| Standard error | 1.78E-18 | 3.9E-18 | 1.60E-18 |

**Automatic parameter extraction**

The parameters of interest, i.e indentation, hysteresis and work of adhesion were calculated by a pyton code written for this purpose. A graphical example of the output for calculation work of adhesion is presented in the figure S2, with the relevant area coloured in blue.


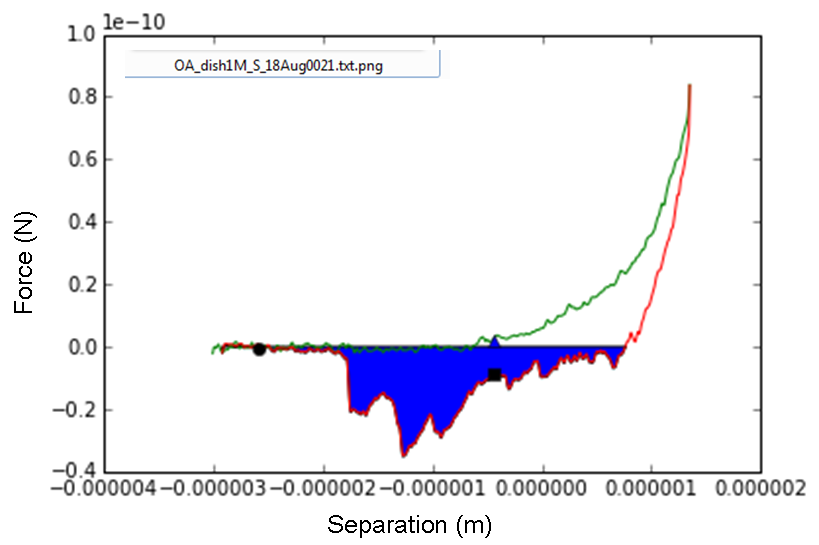


**Figure S2:** A typical plot resulting from automated parameter extraction, blue area marking the area of adhesion.

**References**

1. Ishihara H, McNally DS, Urban JP, Hall AC (1996) Effects of hydrostatic pressure on matrix synthesis in different regions of the intervertebral disk. *J. App. Physiol.* 80:839-846.

2. Hutter JL, Bechhoefer J (1993) Calibration of atomic-force microscope tips. *Rev. Sci. Instrum.* 64(7):1868-1873.

3. Hemmer JD, Dean D, Vertegel A, Langan III E, LaBerge M (2008) Effects of serum deprivation on the mechanical properties of adherent vascular smooth muscle cells. *Proc. IMechE Part H: J. Engineering in Medicine* 222:761-772.

4 Sirghi L, Ponti J, Broggi F, Rossi F. Probing elasticity and adhesion of live cells by atomic force microscopy indentation. *Eur Biophys J Biophy*. 2008;37:935-45.
